# Carbon dioxide levels in neonates: what are safe parameters?

**DOI:** 10.1038/s41390-021-01473-y

**Published:** 2021-07-06

**Authors:** Sie Kei Wong, M. Chim, J. Allen, A. Butler, J. Tyrrell, T. Hurley, M. McGovern, M. Omer, N. Lagan, J. Meehan, E. P. Cummins, E. J. Molloy

**Affiliations:** 1 Department of Paediatrics, School of Medicine, Trinity College, The University of Dublin, Trinity Translational Medicine Institute (TTMI) & Trinity Research in Childhood Centre (TRiCC), Dublin, Ireland; 2 Childrens Hospital Ireland (CHI) at Tallaght, Tallaght University Hospital, Dublin, Ireland; 3 Department of Neonatology, Coombe Women’s and Infant’s University Hospital, Dublin, Ireland; 4 School of Medicine and Conway Institute of Biomolecular and Biomedical Research, University College Dublin, Dublin, Ireland; 5 Neonatology, CHI at Crumlin, Dublin, Ireland

## Abstract

**Abstract:**

There is no consensus on the optimal pCO_2_ levels in the newborn. We reviewed the effects of hypercapnia and hypocapnia and existing carbon dioxide thresholds in neonates. A systematic review was conducted in accordance with the PRISMA statement and MOOSE guidelines. Two hundred and ninety-nine studies were screened and 37 studies included. Covidence online software was employed to streamline relevant articles. Hypocapnia was associated with predominantly neurological side effects while hypercapnia was linked with neurological, respiratory and gastrointestinal outcomes and Retinpathy of prematurity (ROP). Permissive hypercapnia did not decrease periventricular leukomalacia (PVL), ROP, hydrocephalus or air leaks. As safe pCO_2_ ranges were not explicitly concluded in the studies chosen, it was indirectly extrapolated with reference to pCO_2_ levels that were found to increase the risk of neonatal disease. Although PaCO_2_ ranges were reported from 2.6 to 8.7 kPa (19.5–64.3 mmHg) in both term and preterm infants, there are little data on the safety of these ranges. For permissive hypercapnia, parameters described for bronchopulmonary dysplasia (BPD; PaCO_2_ 6.0–7.3 kPa: 45.0–54.8 mmHg) and congenital diaphragmatic hernia (CDH; PaCO_2_ ≤ 8.7 kPa: ≤65.3 mmHg) were identified. Contradictory findings on the effectiveness of permissive hypercapnia highlight the need for further data on appropriate CO_2_ parameters and correlation with outcomes.

**Impact:**

- There is no consensus on the optimal pCO_2_ levels in the newborn.
- There is no consensus on the effectiveness of permissive hypercapnia in neonates.
- A safe range of pCO_2_ of 5–7 kPa was inferred following systematic review.

## Introduction

Carbon dioxide (CO_2_) is a physiological gas produced as a consequence of aerobic metabolism. Plasma CO_2_ levels are tightly regulated under physiological conditions to maintain blood pH. CO_2_ is mainly removed from the body via the lungs and can accumulate in lung pathologies. In humans, normocapnia is defined as a pCO_2_ of between 4.7 and 6.0 kPa (35.3–45.0 mmHg), with pCO_2_ reflecting the balance between CO_2_ production and removal. Consequently, slightly higher pCO_2_ values are associated with the venous circulation as CO_2_-enriched blood is returned towards the lungs.^1^ Importantly, the local microenvironment of cells and tissues can experience pCO_2_ levels that differ markedly from systemic PaCO_2_, e.g., in solid tumours.^2^ This is likely a consequence of local metabolic activity and alterations in local blood supply. Adaptive responses to changes in CO_2_ can be classified as being acute or chronic. Acute responses to CO_2_ are generally sensed through brain stem central chemoreceptors that modulate the rate and depth of breathing to try and maintain normocapnic partial pressures of CO_2_. Chronic responses to CO_2_ can be elicited on a cellular level through CO_2_-dependent changes in gene expression, e.g., genes associated with immune signalling.^3^ The cerebral vasculature is very sensitive to physiological gases. Given the brain’s significant demand for oxygen to maintain normal function, several adaptive cerebral vasodilatory mechanisms are elicited to promote and maintain cerebral blood flow under conditions of hypoxia. Elevated CO_2_ levels, which are frequently associated with hypoxia, also promote an increase in cerebral blood flow through dilation of cerebral arteries and arterioles.^4^ In contrast, hypocapnia leads to vascular constriction and reduced blood flow. Thus, the cerebral vasculature is highly sensitive to the level of circulating physiological gases with hypocapnia and hypercapnia capable of affecting brain oxygen levels indirectly through modulation of cerebral blood flow.

In healthy neonates, the physiological CO_2_ range is defined as 4.7–6.0 kPa (35.3–45.0 mmHg).^5,6^ Given the physiological impact of CO_2_ levels on cerebral vasculature and the impact of CO_2_ on immune signalling, it has been suggested that hyper- and hypocapnia can both have detrimental effects for newborn infants. While CO_2_ monitoring can be undertaken on a continuous basis or intermittently with blood gas measurement, there is currently no consensus on the optimal pCO_2_ levels in the newborn. Similarly, there is limited evidence as to what is clinical best-practice with respect to managing the care of neonates with moderate hypocapnia and hypercapnia.

### Effects of hypocapnia

Hypocapnia in the neonate may be the result of a disease such as transient tachypnoea of the newborn, or more commonly, induced iatrogenically during mechanical ventilation or extracorporeal membrane oxygenation.^5^ Permanent brain injury may be caused by cerebral vasoconstriction and low cerebral tolerance of hypoxia^7,8^ and hypocapnia may worsen ischaemia/reperfusion-induced acute lung injury.^6^ There is a correlation between the degree and duration of hypocapnia and the incidence and severity of these lesions.^7^ In both term and preterm neonates, hypocapnia can therefore be a risk factor for central nervous system damage which may manifest as cerebral palsy (CP), cognitive or developmental disabilities, intraventricular haemorrhage (IVH), periventricular white matter injury and auditory impairment.^5,6,9,10^

### Effects of hypercapnia

Autoregulation of cerebral blood flow and subsequent cerebral oxygenation is undermined when pCO_2_ levels increase.^11^ Severe hypercapnia is of concern due to the risk of cerebral oedema and vasodilation, particularly in relation to infants with neonatal encephalopathy (NE).^12^ High PaCO_2_ predisposes preterm infants to IVH,^9,10,12,13^ and maximum pCO_2_ seems to be an important factor for severe IVH in the first three postnatal days.^14^ Severe hypercapnia alters consciousness and mental state, induces spasms and suppresses cortical activity, which is associated with impaired outcome in preterm infants.^6,15^ There is also a significant association between hypercapnia in low weight infants and bronchopulmonary dysplasia (BPD) as compared to normocapnic controls.^16^

A greater prevalence of necrotising enterocolitis (NEC) was observed in premature neonates with a higher pCO_2_ target (7.3–10.0 kPa = 54.8–75.0 mmHg) as compared to the normal target group.^17^ Neonatal survival rate was associated with the level of PaCO_2_ in patients with congenital diaphragmatic hernia (CDH). Infants with CDH that remained hypercapnic post-resuscitation (9.6 ± 2.5 kPa = 72.0 ± 18.8 mmHg) had a worse prognosis as compared to those who were normocapnic.^18^ However, it is important to note that this observation may be explained by the severity of lung hypoplasia, with more severe hypoplasia manifesting as persistent hypercapnia.

The need for mechanical ventilation in the neonatal period has been associated with lung injury and long-term respiratory morbidity such as BPD.^19,20^ There is also a higher risk of premature brain injury such as periventricular leukomalacia (PVL) and IVH associated with its use.^10,21^ The duration and intensity of ventilation has been implicated in the pathogenesis of neonatal lung injury^22^ with large tidal volumes and resultant volutrauma being especially damaging to the immature lung.^22^ Permissive hypercapnia is a ventilatory strategy that permits relatively high levels of CO_2_ in ventilated neonates, thereby allowing lower tidal volumes to be used in patients who are mechanically ventilated. This less aggressive approach to ventilation reduces the risk of volutrauma in ventilated neonates and may improve respiratory outcomes and survival rates. Effective CO_2_ elimination can occur at lower tidal volumes and peak inspiratory pressures as increased PaCO_2_ achieved using permissive hypercapnia increases CO_2_ elimination for the same minute ventilation (from the equation *k* × VCO_2_ = PaCO_2_ × Va, with Va representing alveolar ventilation). Hypercapnic acidosis also improves ventilation–perfusion mismatch and allows greater unloading of O_2_ at the tissues (Bohr effect). In addition, there is also an increased respiratory drive to reduce apnoea and increased cardiac output.^23,24^ Thus, it has been widely employed in the ventilation of preterm infants. We aimed to determine safe pCO_2_ levels in neonates with reference to the potential side effects of hypercapnia and hypocapnia identified within this population.

## Methods

### Literature search

The methodology of this systematic review was designed and conducted in accordance with the Preferred Reporting Items for Systematic Reviews and Meta-Analyses (PRISMA) statements^25^ and guidelines from the Meta-analysis of Observational Studies in Epidemiology (MOOSE).^26^ A literature search was done on Pubmed, Embase and Scopus and the terms undertaken were: “neonates” AND (“hypocapnia” OR “hypercapnia”). Additional studies identified during a manual search were also included.

### Inclusion and exclusion criteria

Studies that satisfied the following criteria were included into our literature review: (1) full text available in English, (2) human subjects and (3) peer-reviewed journals. Exclusion criteria were established to eliminate studies beyond the scope of the review: editorials; letters and case reports; duplicate publications; and use of animal models. The publication years were not restricted due to a limitation in the number of studies available.

### Quality assessment and data extraction

In the first phase of selection, the titles and abstracts were reviewed by two independent reviewers to determine their relevance. Disagreements were either resolved by a third reviewer or settled by consensus. The second phase of selection involved full text screening where an independent reviewer determined their eligibility. Covidence online software^27^ was employed in both phases of screening to streamline the relevant articles. Data extracted includes (1) side effects of hypercapnia, hypocapnia and permissive hypercapnia(2) pCO_2_, PaCO_2_ or PcCO_2_ levels and (3) population characteristics. Analysis was subsequently performed, and results were tabulated according to preterm and term termed neonates (Tables 1 and 2). As safe pCO_2_ ranges were not explicitly concluded in the studies chosen, it was indirectly extrapolated with reference to pCO_2_ levels that were found to increase the risk of neonatal disease. The data were then categorised based into pre-term termed and term termed infants. The quality of the studies was assessed using the hierarchy of evidence.^28^

**Table 1:** Limits of CO_2_: hypercapnic, hypocapnic and permissive hypercapnia parameters for preterm infants (BPD, IVH, PVL, CP).

Pathology	Citation	Study type	Safe range	Comments
BPD	Subramanian et al.,^16^ *n* = 425	Observational cohort multicentre study (Level 4)	pCO_2_ < 6.67 kPa (<50.0 mmHg)	May reduce the risk of BPD in low and extremely low birth weight infants
	Mariani et al.,^22^ *n* = 49	RCT (Level 3)	PaCO_2_ 6.0–7.3 kPa (45.0–54.8 mmHg)	May be a viable alternative to normocapnia in extremely preterm neonates treated with surfactant
	Thome et al.^31^ (2018), *n* = 359	Exploratory analysis of a RCT (Level 3)	pCO_2_ < 7.33 kPa (<55.0 mmHg)	Hypercapnia significantly increases mortality and incidence of BPD of extremely low birth weight infants
	Carlo et al.^28^ (2002), *n* = 220	RCT (Level 3)	PaCO_2_ < 6.8 kPa (<51.0 mmHg)	Does not decrease mortality and incidence of BPD in extremely low weight infants, however, use of mechanical ventilation at 36 weeks is reduced
	Ambalavanan N et al.,^30^ *n* = 1316	Secondary analysis of a RCT (Level 3)	PaCO_2_ 6.0–7.3 kPa (45–55 mmHg)	Hypercapnia (higher maximum, average or fluctuation) significantly increases mortality and risk of BPD of extremely low birth weight infants
IVH	Ambalavanan N et al.,^30^ *n* = 1316	Secondary analysis of a RCT (Level 3)	PaCO_2_ 6.0–7.3 kPa (45–55 mmHg)	Higher maximum PaCO_2_ is associated with higher risk of IVH and mortality in extremely low birth weight infants independently
	Zayek et al.^28^ (2014) *n* = 580	Retrospective cohort study (Level 4)	pCO_2_ < 6.0 kPa (<45.0 mmHg)	May lower the risk of IVH in extremely low birth weight infants
	Vela-Huerta et al.^29^ (2009) *n* = 83	Retrospective case control study (Level 5)	PaCO_2_ < 7.3 kPa (<45.0 mmHg)	May lower the risk of severe IVH in extremely low birth weight infants
	Köksal et al.^30^ (2002) *n* = 120	Prospective cohort study (Level 4)	PaCO_2_ < 8.0 kPa (<60.0 mmHg)	May decrease the risk of IVH in very low and extremely low birth weight premature infants
	Waitz et al.,^14^ *n* = 279	Retrospective cohort study (Level 4)	PaCO_2_ 5.3–7.7 kPa (39.8–57.8 mmHg)	Moderate permissive hypercapnia (5.3–7.7 kPa or 39.8–57.8 mmHg) is possible in extremely preterm neonates with GM-IVH Avoid PaCO_2_ levels >7.7 kPa (or >57.8 mmHg) as increases risk IVH
	Fabres et al.,^10^ *n* = 849	Retrospective cohort study (Level 4)	PaCO_2_ 5.2–8.0 kPa (39.0–60.0 mmHg)	The suggested optimum PaCO_2_ range in very low and extremely low birth weight and extremely preterm babies. Extreme PaCO_2_ values (>60 mmHg) should also be avoided in this group
PVL	Liu et al.,^5^ *n* = 921	Prospective cohort study (Level 4)	PaCO_2_ > 4.67 kPa (>35.0 mmHg)	Significant increase in incidence of PVL in premature infants that are hypocapnic (PaCO_2_ ≤ 4.67 kPa or ≤35.0 mmHg)
Neurodev & CP	Thome et al.^40^ (2017) *n* = 359	RCT (Level 3)	PaCO_2_ < 7.3 kPa (<54.8 mmHg)	May decrease the risk of mortality and neurodevelopmental impairment
	Brown et al.,^9^ *n* = 147	Secondary analysis RCT (Level 3)	PaCO_2_ 6.0–6.7 kPa (45.0–50.3 mmHg)	May be safe neurologically. PaCO_2_ should be <7.0 kPa (or 52.5 mmHg) as PaCO_2_ above 7.0 kPa (or 52.5 mmHg) is associated with severe IVH and death
	Collins et al. ^25^ (2001), *n* = 657	Prospective cohort study (Level 4)	PaCO_2_ > 4.7 kPa (>35.3 mmHg)	Recommended avoiding PaCO_2_ levels <4.7 kPa (or <35.3 mmHg) during mechanical ventilation in very low birth weight infants

1 kPa = 7.5 mmHg = 7.5 torr = 10.2 mm H_2_O; *BPD* bronchopulmonary dysplasia, *IVH* intraventricular haemorrhage, *GM-IVH* germinal matrix-intraventricular haemorrhage, *PVL* periventricular leukomalacia, *CP* cerebral palsy.

**Table 2:** Limits of CO_2_: hypercapnic, hypocapnic and permissive hypercapnia parameters for term infants (NE and CDH).

Pathology	Citation	Study type	Safe range	Comments
NE	Nadeem et al.^33^ (2009), *n* = 55	Retrospective cohort study (Level 4)	pCO_2_ 2.6–3.3 kPa (19.5–24.7 mmHg)	No significant association between moderate hypocapnia (2.6–3.3 kPa or 19.5–24.7 mmHg) and hypercapnia (>6.6 kPa or 49.5 mmHg) over the first 3 days after birth and adverse neurodevelopmental outcomes
	Pappas et al.^34^ (2011), *n* = 204	Secondary study RCT (Level 3)	pCO_2_ > 2.6–3.3 kPa (>19.5–24.7 mmHg)	Association between poor neurodevelopmental outcomes at 18–22 months of age and both minimum pCO_2_ and cumulative 2.6–3.3 kPa (or 19.5–24.7 mmHg) in infants with NE
	Klinger et al.^54^ (2005), *n* = 244	Retrospective cohort study (Level 4)	PaCO_2_ > 2.6 kPa >19.5 mmHg)	Severe hypocapnia (PaCO_2_ < 2.6 kPa or <19.5 mmHg) is associated with adverse neurological outcomes (severe CP, etc.) in term infants with post-asphyxial neonatal encephalopathy.
CDH	Abbas PI et al.,^18^ *n* = 74	Retrospective cohort study (Level 4)	PaCO_2_ 5.2 kPa (39.0 mmHg)	Maintaining initial, best PaCO_2_ and PaCO_2_ after resuscitation within physiological range (5.2 kPa or 39.0 mmHg) is associated with improved survival rates
	Bojanic et al.^36^ (2015), *n* = 83	Retrospective cohort study (Level 4)	PcCO_2_ ≤ 8.7 Pa (≤65.3 mmHg)	Permissive hypercapnia (PcCO_2_ ≤ 8.7 kPa or ≤65.3 mmHg) is potentially protective

1 kPa = 7.5 mmHg = 7.5torr = 10.2 mmH_2_O, *NE* neonatal encephalopathy, *CDH* congenital diaphragmatic hernia.

## Results

**Fig. 1:**
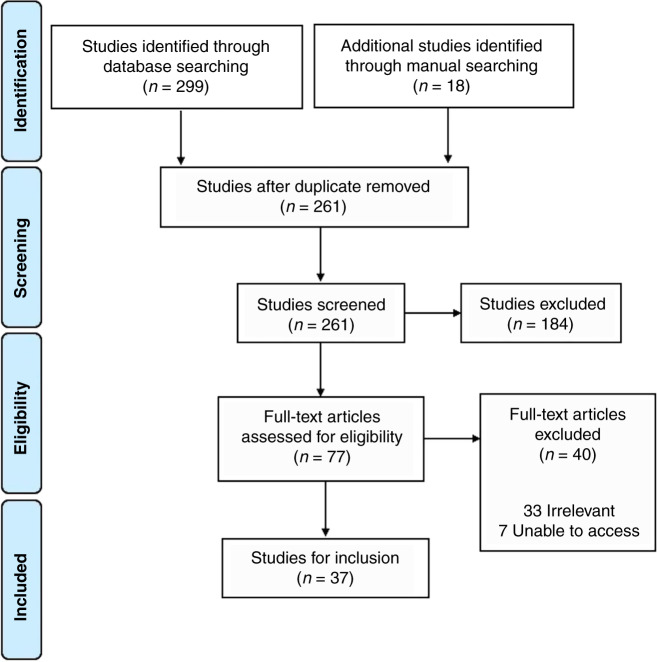
PRISMA flowsheet: studies of Carbon Dioxide in Neonates.

Two hundred and ninety-nine studies were identified via database searching and 18 additional studies were identified through manual searching. There were 261 papers after duplicates were removed. Seventy-seven full-text articles remained after the first round of screening and 37 studies were included in this review (Fig. 1).

### Preterm infants

#### Hypocapnia

Brown et al. reviewed the range of permissive hypercapnia used in clinical practice and determined the relationship between PaCO_2_ and pH during the first three days of life and negative health outcomes. Their subjects were 147 premature neonates who were less than 32 weeks gestational age. Preterm infants of less than 29 weeks gestational age had a higher risk of severe IVH and PVL if their PaCO_2_ fell below 4.0 kPa (30.0 mmHg) within the first two days of life. Similarly, if the same group of infants had at least 3 PaCO_2_ values less than 4.0 kPa (30.0 mmHg) within the first day of life they were at increased risk of BPD (*p* = 0.036).^9^

Liu et al.^5^ prospectively found a significantly higher incidence of PVL in premature newborns with hypocapnia (PaCO_2_ ≤ 4.67 kPa = 35.0 mmHg) as compared to the preterm control group, suggesting that hypocapnia is an important high-risk factor for PVL. A prospective study by Collins et al. which involved 1105 infants with birth weight between 500 and 2000 g found that the unadjusted univariate rates of disabling CP within ventilated infants by quintile of cumulative hypocapnia exposure has a statistically significant linear trend. It also found that with cumulative hypocapnia (pCO_2_ < 4.7 kPa = 35.3 mmHg) or prolonged ventilation as a ventilatory risk factor, the risk of CP increased by 7–9-fold as compared to ventilated newborns without any risk factors. In multivariate analysis of the same research, the only ventilation-associated risk factor that is statistically significant regardless of the multivariate model in which it is placed is cumulative hypocapnia.^29^

#### Hypercapnia

A randomised controlled trial carried out by Carlo et al among 220 extremely low birth weight infants found that permissive hypercapnia (PaCO_2_ > 6.93 kPa = 52.0 mmHg) did not significantly decrease mortality or incidence of BPD (*p* = 0.43). However, the use of mechanical ventilation at 36 weeks is significantly lesser in the minimally ventilated group as compared to the routine ventilation group (*p* < 0.01). This indicates that permissive hypercapnia might lower the severity of lung injury but not the incidence.^29,30^ Subramanian et al. conducted a multicentre observational cohort study of 425 newborns in Columbia, which examined the rates of BPD in low and extremely low birth weight infants (500–1499 g). They described a strong association between hypercapnia (PaCO_2_ > 6.67 kPa = 50.0 mmHg) and BPD in the first few days of life. This was based on the observation that the incidence of BPD was greater as compared to the group without hypercapnia (*p* = 0.024). The risk of BPD was increased even with permissive hypercapnia.^16^ In a secondary exploratory data analysis on 1316 extremely preterm infants of extremely low birth weight from the large multicenter trial Surfactant, Positive Pressure, and Oxygenation Randomised Trial (SUPPORT), Ambavlanan et al.^31^ found that higher maximum, average and fluctuation of PaCO_2_ correlated with an increased risk of BPD or death (*p* = 0.0002). Thome et al.^32^ found that mortality and incidence of moderate-to-severe BPD were significantly raised in hypercapnic extremely low birth weight premature newborns (*p* < 0.01) in a multicentre study. In concordance with these results, a retrospective cohort study of 268 extremely premature infants of very low and extremely low birth weight observed higher rates of severe IVH in infants with larger pCO_2_ fluctuations (*p* = 0.02).^33^

Ambalavanan et al.^31^ in the secondary analysis of the SUPPORT trial (a large multicentre trial) found that higher peak pCO_2_ correlated with an increased risk of severe IVH/death (*p* = 0.029) and neurological impairment/death even at lower ranges of hypercapnia. Higher maximum PaCO_2_ was an independent risk factor for severe IVH/death even after statistical adjustment for illness severity.^30^^,^^31^ A retrospective cohort study by Zayek et al. investigated the role of mild to moderate hypercapnia (6.0–8.0 kPa = 45.0–60.0 mmHg) and acidaemia in the occurrence of severe IVH in 580 extremely low birth weight infants within the first 48 h of life. They found that higher pCO_2_ (PaCO_2_ > 8.0 kPa = 60.0 mmHg) was significantly associated with increased rates of severe IVH as compared to normocapnic infants.^34^ Vela-Huerta et al.^35^ conducted a single centre retrospective case–control study on 28 cases of extremely low birth weight preterm infants with grade III or IV IVH and 55 controls and found that PaCO_2_ > 7.3 kPa (>54.8 mmHg) was only associated with grade IV IVH. Köksal et al.^36^ found hypercapnia (pCO_2_ > 8.0 kPa or >60.0 mmHg) to be a risk factor for IVH (*p* < 0.0001) in 120 premature infants with very low and extremely low birth weight. In a retrospective study, Fabres et al. established that extreme PaCO_2_ levels and the magnitude of pCO_2_ fluctuation were good indicators of severe IVH in very low and extremely low birth weight preterm babies. They have also identified that maximal PaCO_2_ > 8.0 kPa (>60.0 mmHg) and time-weighted PaCO_2_ > 7.0 kPa (>52.5 mmHg) are associated with severe IVH. The optimal range of pCO_2_ derived by the team was 5.2–8.0 kPa (39.0–60.0 mmHg) and at that range, there was only a 3% incidence of severe IVH.^10^

Waitz et al. explored the risk factors of IVH in extremely preterm neonates. Higher maximal PaCO_2_ was a risk factor for IVH (*p* = 0.001) and the risk increased by a factor of 1.04 for every 0.13 kPa (0.98 mmHg).^14^ The same study further quantified that moderate permissive hypercapnia (7.3–8.7 kPa = 54.8–65.2 mmHg) was not associated with a higher incidence of severe IVH when compared to the normocapnic group (5.3–7.7 kPa = 39.8–57.8 mmHg). Ali et al.^37^ stated that there was a significant independent association between hypercapnia and the development of Retinpathy of prematurity (ROP), making hypercapnia a significant variable of development of ROP.

### Neonatal encephalopathy

#### Hypocapnia

Lopez Laporte et al. studied 198 newborns exposed to asphyxia and found a possible association between hypocapnia and the severity of the brain injury. They also highlighted that “asphyxiated” newborns treated with ventilation and hypothermia have higher incidences of brain injury.^13^

Moderate hypocapnia (2.6–4.7 kPa = 19.5–35.3 mmHg) was not found to be a significant risk factor of severe disability and death in NE by Nadeem et al.^38^ or Hansen et al.^39^ Hansen et al. found no effect of moderate, severe hypocapnia or time-weighted cumulative hypocapnia on neurodevelopmental outcomes. However, Hansen and co-workers suggested that neonates with NE who are treated with therapeutic hypothermia should not be over-ventilated to prevent hypocapnia and its adverse effects.^38,39^

Pappas et al. found that minimum pCO_2_ defined as isolated severe hypocarbia, cumulative pCO_2_ < 4.7 kPa (35.3 mmHg) and larger pCO_2_ fluctuations in the first 16 h of life were associated with disability and death at 18–22 months in neonates that have NE. The greater the cumulative exposure, the worse the outcome and minimum pCO_2_ was the only significant predictor of negative outcome.^40^

Consistent with the findings by Pappas et al., Linggapan et al. suggested that probability of an unfavourable outcome increases as pCO_2_ values decrease. In newborns with severe NE, hypocapnia (pCO_2_ between 2.7 and 5.3 kPa = 20.3–39.8 mmHg) was associated with a higher probability of unfavourable outcome as compared to those with moderate NE.^4^

#### Hypercapnia

A retrospective cohort study by Lopez Laporte^13^ et al. found that neither the magnitude of CO_2_ fluctuations nor hypercapnia were significant risk factors for brain injury in NE. Hypercapnia was not a significant risk factor for severe disability and death in infants with NE.^38^ A similar relationship was also illustrated in a study by Pappas et al.,^40^ which reported that maximum pCO_2_ and cumulative exposure to hypercapnia were not significant predictors of disability or death. Although NE infants with cumulative pCO_2_ above the 50th percentile were more likely to have seizures and needed more time to achieve spontaneous respiration, only the time to spontaneous respiration of more than 10 min had a significant association with adverse outcomes.^41^ Hansen et al.^39^ retrospectively studied 23 neonates with moderate to severe NE treated with 72 h of hypothermia and it was found that those from the group with adverse neurodevelopmental outcomes had greater PaCO_2_ variability than those from the group with favourable neurodevelopmental outcomes. The hypoxic-ischaemic encephalopathy therapy optimization in neonates for better neuroprotection with inhaled CO_2_ (HENRIC) study, which involved 10 term infants, recently demonstrated that inhaled 5% CO_2_ was feasible and safe for correcting hypocarbia in NE.^41^

#### Congenital diaphragmatic hernia

CDH is associated with pulmonary hypoplasia and respiratory distress at birth and infants with CDH frequently require mechanical ventilation following delivery. Abbas et al. found that neonates with CDH who remained hypercapnic (PaCO_2_ > 9.6 kPa > 72.0 mmHg) had poorer survival rates than those who were normocapnic (PaCO_2_ = 5.2 kPa = 39.0 mmHg) after resuscitation (*p* < 0.001). However, hypercapnia may simply be a marker of more severe lung hypoplasia, thus explaining the increased mortality in this group. The study also investigated the impact of initial PaCO_2_ and best PaCO_2_ on survival rates and patients with an initial and best PaCO_2_ of >8.0 kPa (>60.0 mmHg) had higher mortality rates (*p* = 0.003 and *p* = 0.001 respectively). Best PaCO_2_ and PaCO_2_ after resuscitation are better predictors of neonatal survival compared to foetal lung volumes. A retrospective study by Bojanic et al. showed that survival rates were higher among term neonates with CDH who were treated according to a permissive hypercapnia protocol (*p* = 0.019). This study also observed that those with higher end-capillary partial pressure (PcCO_2_) on admission had a lower probability of survival (*p* = 0.008), which raises the possibility of PcCO_2_ on admission being a good marker of survival prognostication.^42^

#### Permissive hypercapnia

A meta-analysis by Ma et al.^38^ concluded that permissive hypercapnia did not have notable effects on IVH, PVL, NEC, ROP, mortality or air leaks in extremely low birth weight infants. Permissive hypercapnia also did not lead to elevated risks of CP, visual or hearing impairment nor did it decrease the risk of BPD.^43^

A 2001 Cochrane review published by Woodgate and Davies included two randomised controlled trials.^22^ This review concluded that evidence did not support the use of permissive hypercapnia to prevent morbidity and mortality in newborns receiving mechanical ventilation, particularly BPD or death at 36 weeks, severe IVH and PVL. Although Brown et al. did not find a significant reduction in BPD in secondary analyses of randomised controlled trials when neonates were treated with mild hypercapnia (6.0–7.2 kPa = 45.0–54.0 mmHg), a mean PaCO_2_ of 7.0 kPa (52.5 mmHg) was found to be associated with severe IVH or death.^9^ Thome et al.^44^ found PaCO_2_ levels of 7.3–8.7 kPa (54.8–65.3 mmHg) were associated with higher mortality, neurodevelopmental impairment, and an increase in the combined outcome of mental impairment or death and higher PaCO_2_ levels in the first 3 days of life were associated with IVH by Waitz et al.^14^

## Discussion

Different definitions of hypo- and hypercapnia make it challenging to determine a standardised range of CO_2_ in neonates.^45^ Avoiding both hypocapnia and hypercapnia is optimal but there is not a definite range of optimal CO_2_. However, permissive hypercapnia within a limited range may be beneficial as higher alveolar CO_2_ increases CO_2_ elimination and stimulates the neonate’s respiratory drive which facilitates weaning from mechanical ventilation.^7^ Similarly, severe hypercapnia should be avoided as it correlates with a higher IVH incidence.^34,35,42^ pCO_2_ fluctuations may be more significantly associated with severe IVH than the mere presence of hypercapnia.^31^ Extremely preterm and very low birth weight infants may benefit from moderate permissive hypercapnia as it was not found to increase the incidence of IVH.^14,46^

### Hypocapnia

Hypocapnia is detrimental and contributes to PVL development as it worsens cerebral blood flow dysregulation and exacerbate hypoxic-ischaemic white matter damage.^5^ Neonates are particularly vulnerable to hypocapnia at specific time periods and immaturity of respiratory control in the neonatal period could play a role^47,48^ and hypocapnia is associated with an increased risk of CP in ventilated low birth weight infants. Therefore, avoiding pCO_2_ < 4.7 kPa (35.3 mmHg) has been recommended as values below this were associated with brain injury (35.3 mmHg).^29^

Infants with NE may be able to tolerate hypercapnic states as it has not been shown to contribute to increased morbidity and mortality.^13,40^ NE is frequently associated with hypocapnia.^4^ Hypocapnia increases lactate production (secondary to ischaemia from cerebral vasoconstriction), cerebral oxygen demand and neuronal excitability. Hypocapnia^4^ and PaCO_2_ variability^38^ are modifiable risk factors that may potentially help to improve the neurological outcomes. Therefore, a strategy aimed at lowering the risk of prolonged and severe hypocapnia are CO_2_ inhalation as described in the HENRIC study is attractive.^41^ Inconsistent results exist for CDH, with Abbas et al.^18^ recommending normocapnia to optimise survival and Bojanic et al.^42^ proposing that permissive hypercapnia is protective. However, there is a paucity of data in this area and long-term effects and outcomes are unknown in children with CDH related to PaCO_2_ and the evidence from preterm data suggest early hypercapnia may be detrimental.

For hypocapnia, reported parameters for CP (pCO_2_ < 4.7 kPa; <35.3 mmHg), NE (PaCO_2_ < 3.3 kPa; <24.7 mmHg) and PVL (PaCO_2_ < 4.67 kPa; <35.0 mmHg) were identified. For hypercapnia, parameters mentioned for BPD (pCO_2_ > 6.67 kPa; >50.0 mmHg), IVH (PaCO_2_ > 7.7–8.0 kPa; >57.8–60.0 mmHg) and CDH (PaCO_2_ > 5.2 kPa; >39.0 mmHg and PcCO_2_ ≥ 8.7 kPa; ≥65.3 mmHg) were described.

### Hypercapnia

Permissive hypercapnia has not been validated nor have the safe upper limit of permissive hypercapnia been defined. Permissive hypercapnia is thought to reduce the risk of BPD and confer protection against volutrauma^9^ although results are equivocal. A randomised trial by Thome et al.^17^ found that BPD rates did not improve with moderate hypercapnia. In this trial^17^ higher permissive hypercapnia was compared to lower permissive hypercapnia with no normocapnic study group showing that higher pCO_2_ targets did not increase any benefit over mild permissive hypercapnia

A follow-up study by Thome et al.^49^ concluded that a higher PaCO_2_ target did not affect neurodevelopmental outcomes in extremely preterm infants. Moreover, Subramanian et al.^16^ suggested that permissive hypercapnia predisposes an infant to BPD. A possible explanation for this phenomenon could be that the benefits of low tidal volume may be offset by higher ventilator rates, thereby minimising the advantage of minimal ventilation in extremely preterm neonates.^17^ Not only does severe IVH and retinopathy not improve with moderate hypercapnia,^17^ it may actually increase the risk of IVH. A suggested mechanism is increased cerebral blood flow secondary to hypercapnia which leads to cerebral oedema, raised intracranial pressure and resultant haemorrhage.^50^ However, recent literature suggests that it is the ischaemia, secondary to hypocapnia, that causes a haemorrhage or an extension of the pre-existing haemorrhage following reperfusion of the ischaemic region. The length of ventilation has also been implicated in the effectiveness of permissive hypercapnia. Ryu et al.^23^ reviewed three trials and concluded that only neonates who received longer intervention (10 days) had reduced rates in BPD whilst shorter interventions yielded heterogeneous results. Extreme hyper- or hypocapnia and rapid fluctuations in pCO_2_ were detrimental to preterm infants especially in the first week of life. The optimal pCO_2_ goal in clinical practice is still undetermined, but tolerating modest hypercapnia (pCO_2_ < 6.7 kPa [50 mmHg]) may be beneficial.

CO_2_ levels are known to influence immune and inflammatory signalling in cells, tissues, animals and humans. Hypercapnia can be either protective or deleterious depending on the context. The current view is that hypercapnia is harmful in the context of infection but may in fact be beneficial in the context of uncontrolled inflammation.^1^ Clinically, hypercapnia is associated with a higher hazard ratio for death in chronic obstructive pulmonary disease,^51^ higher ICU mortality in acute respiratory distress syndrome^52,53^ among adults. Given these adverse clinical outcomes, it may be surprising that hypercapnia is proposed to have anti-inflammatory effects and indeed was found to be protective in a recent clinical trial of “therapeutic hypercapnia” in single lung lobectomy patients.^54^ The molecular mechanisms underpinning CO_2_-dependent suppression of immune signalling are not fully elucidated but likely involve suppression of pro-inflammatory signalling cascades. The master-regulator of immune and inflammatory signalling, nuclear factor κB (NFκB), has been implicated in both hypercapnia and hypercapnic acidosis. Cummins et al.^55,56^ have reported CO_2_-dependent modulation of the NFκB pathway on multiple levels including protein localisation, transcriptional activation and protein–protein interactions. The concept of NFκB sensitivity to CO_2_ levels is additionally supported by several studies from the Laffey lab^57,58^ and others.^59^ While CO_2_-dependent modulation of NFκB signalling is important in several cell types it is likely that other factors are also involved alone or in combination with NFκB, including HSF-1,^60^ FOXO3a^61^ and CREB.^62^ Thus, CO_2_ represents a potentially modifiable factor with the potential to suppress damaging inflammatory signalling in the lung. However, this benefit does not extend to neonates who have sustained ventilator-associated injuries as hypercapnic acidosis impedes plasma membrane repair.^61,63^

This review was limited by the research design utilised by the studies included. Some studies comprised small cohorts with multiple clinical morbidities making it statistically underpowered and thus cannot make associations or establish causal relationships. The retrospective nature of some studies also affects the credibility of the information presented. Different ventilation settings and different sources of blood gas samples (e.g. arterial, capillary and venous) across the cohort can affect the accuracy of the results obtained. For example, capillary and venous samples may artificially increase pCO_2_ values and overestimate the effects hypocapnia and hypercapnia have on neonates. Different CO_2_ measurement methods (e.g. blood gas, capnography) also contribute to variation in the results obtained. Lastly, fixed interval monitoring rather than continuous monitoring may overlook important CO_2_ trends.

Based on the literature review performed, it appears that pCO_2_ levels of approximately 5–7 kPa (37.5–52.5 mmHg) may be safe for neonates requiring ventilatory support. Intervention to therapeutically alter carbon dioxide has been suggested in the recent HENRIC study which demonstrated the use of CO_2_ insufflation to prevent hypocarbia in NE. While the research for safe O_2_ ranges in preterm and term infants is well underway, the same advancement is required for CO_2_. Contradictory findings highlight the need for rigorous evidence to establish the role of permissive hypercapnia in clinical practice and if beneficial, the safe upper limit of permissive hypercapnia. Until safe ranges have been clearly defined, this ventilation strategy should be used with caution.
